# Uptake of Newly Licensed Influenza Vaccine Formulations Among Patients Receiving Chronic Hemodialysis During the 2010/2011 to 2021/2022 Influenza Seasons

**DOI:** 10.1016/j.xkme.2026.101243

**Published:** 2026-01-06

**Authors:** John M. Sahrmann, J. Bradley Layton, Katelin B. Nickel, John W. Davis, Vikas R. Dharnidharka, David J. Weber, Anne M. Butler

**Affiliations:** 1 Division of Infectious Diseases, Department of Internal Medicine, Washington University School of Medicine, St. Louis, MO; 2 RTI Health Solutions, Durham, NC; 3 Department of Internal Medicine, Washington University in St. Louis/Barnes-Jewish Hospital, St. Louis, MO; 4 Department of Pediatrics, Robert Wood Johnson Medical School at Rutgers University, New Brunswick, NJ; 5 Division of Infectious Diseases, Department of Medicine, University of North Carolina at Chapel Hill, Chapel Hill, NC

To the Editor:

Influenza causes substantial morbidity and mortality in patients with kidney failure. The Centers for Disease Control and Prevention (CDC) has long recommended that patients with kidney failure receive annual influenza vaccination^1^ but does not state a preferred vaccine formulation. Recently, the CDC preferentially recommended vaccination with enhanced formulations in persons aged ≥65 years including high-dose inactivated influenza vaccine (hdIIV), adjuvanted inactivated influenza vaccine (aIIV), or recombinant influenza vaccine (RIV) vaccine rather than standard-dose, egg-based, inactivated influenza vaccine (sdeIIV). sdeIIV elicits inferior immunogenicity responses in patients with kidney failure relative to the general population and may be inferior in effectiveness to more novel formulations.^2^, ^3^, ^4^ Several enhanced influenza vaccine formulations were introduced in the United States starting in 2009, including hdIIV, aIIV, RIV, and cell culture–based (ccIIV). Before 2013, sdeIIV accounted for most influenza vaccination among patients with kidney failure.^5^ Otherwise, little is known about utilization of these novel formulations in patients with kidney failure. We sought to evaluate utilization of influenza vaccine formulations from the 2010/2011 to 2021/2022 seasons in a national cohort of patients ≥65 years undergoing hemodialysis.

We used data (including Medicare claims) from the United States Renal Data System (USRDS),^6^ a government-funded data source including most individuals with kidney failure in the United States. For each influenza season (2010/2011 to 2021/2022; Table S1), we identified a cohort of adults alive on August 1, aged ≥65 years, with Medicare as primary payer, and receiving chronic, in-center hemodialysis for ≥3 months before vaccination. We identified the first influenza vaccination from August 1 to end of influenza season (or December 31, 2021, because of end of available data; Table S2). Patients were eligible for multiple seasons. We assessed demographic and clinical characteristics, comorbid conditions, frailty indicators, preventive health services, and other health care utilization (Table S3).

**Figure 1:**
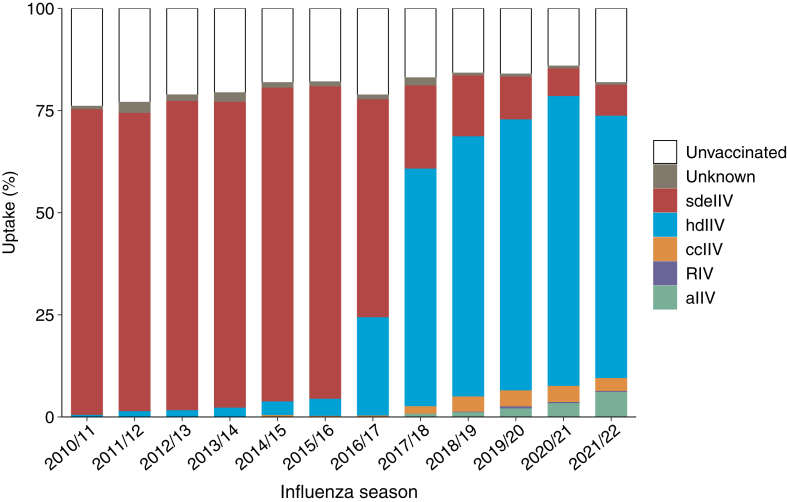
Uptake of influenza vaccine formulations among patients with kidney failure receiving chronic hemodialysis, during the 2010/2011 to 2021/2022 influenza seasons in the United States.

We identified 400,238 eligible patients who contributed 1,190,564 unique seasons (mean age, 74.6 years; 50.7% male; 60.9% White race; 32.0% Black race; 37.4% Medicaid dual-eligibility). The distribution of influenza vaccine formulations was as follows: sdeIIV (46.3%), hdIIV (31.0%), ccIIV (1.5%), aIIV (1.1%), RIV (0.2%), unknown (1.1%), and unvaccinated (18.8%) (Table 1). Initially, sdeIIV use was predominant; however, use markedly declined from 2010/2011 (74.8%) to 2021/22 (7.6%) (Fig 1). Simultaneously, hdIIV use increased considerably from 2010/2011 (0.6%) to 2021/2022 (64.2%). Particularly notable shifts from sdeIIV to hdIIV occurred in the 2016/2017 and 2017/2018 seasons. By the 2021/2022 season, patients receiving dialysis predominantly received hdIIV, whereas 9.2% received either aIIV or ccIIV. Formulation-specific uptake increased to as high as 6.0% for aIIV in 2021/2022, 3.9% for ccIIV in 2018/2019, and 0.7% for RIV in 2019/20. The proportion of unvaccinated patients decreased from 2010/2011 (24.0%) to 2020/2021 (14.1%), followed by a small increase in 2021/2022 (18.2%). Among all vaccine recipients, ccIIV, aIIV, and RIV recipients were the most likely to reside in the Northeast, and recipients of aIIV and RIV were most likely to attend a nonprofit dialysis clinic and not be dual-eligible for Medicaid (Tables 1 and S4). Unvaccinated individuals generally had lower prevalence of comorbid conditions and preventive health services versus vaccinated patients. Table S5 presents demographic characteristics by season and formulation type.

**Table 1:** Select Demographic and Clinical Characteristics of Patients with Kidney Failure Receiving Chronic Hemodialysis During the 2010/2011 to 2021/2022 Influenza Seasons in the United States, by Influenza Vaccine Formulation.^a^^,^^b^

Characteristic	sdeIIV (n = 551,811; 46.3%)	hdIIV (n = 368,544; 31.0%)	ccIIV (n = 18,211; 1.5%)	aIIV (n = 12,761; 1.1%)	RIV (n = 2,339; 0.2%)	Unknown (n = 12,996; 1.1%)	Unvaccinated (n = 223,902; 18.8%)
Demographic characteristics							
Age, mean (SD)	75 (7.0)	75 (7.0)	75 (7.2)	75 (7.1)	75 (7.1)	75 (7.1)	74 (7.0)
Male	278,095 (50.4)	191,892 (52.1)	9,387 (51.5)	7,024 (55.0)	1,285 (54.9)	6,566 (50.5)	108,832 (48.6)
Race							
White	338,731 (61.4)	233,124 (63.3)	10,635 (58.4)	8,532 (66.9)	1,579 (67.5)	8,098 (62.3)	124,707 (55.7)
Black	173,413 (31.4)	108,200 (29.4)	5,670 (31.1)	2,906 (22.8)	625 (26.7)	3,762 (29.0)	86,034 (38.4)
Other	39,667 (7.2)	27,220 (7.4)	1,906 (10.5)	1,323 (10.4)	135 (5.8)	1,136 (8.7)	13,161 (5.9)
Residence in metropolitan statistical area	485,776 (88.0)	323,259 (87.7)	16,837 (92.5)	11,804 (92.5)	2,046 (87.5)	11,494 (88.4)	199,090 (88.9)
Region							
Northeast	94,275 (17.1)	60,763 (16.5)	5,490 (30.1)	3,044 (23.9)	730 (31.2)	3,468 (26.7)	44,003 (19.6)
South	243,746 (44.2)	162,305 (44.0)	6,826 (37.5)	4,821 (37.8)	960 (41.0)	4,513 (34.7)	96,341 (43.0)
West	104,414 (18.9)	71,315 (19.4)	3,377 (18.5)	2,902 (22.7)	277 (11.8)	2,891 (22.2)	39,050 (17.4)
Midwest	109,376 (19.8)	74,161 (20.1)	2,518 (13.8)	1,994 (15.6)	372 (15.9)	2,124 (16.3)	44,508 (19.9)
Dual-eligible for Medicaid	206,367 (37.4)	129,196 (35.1)	7,344 (40.3)	3,945 (30.9)	722 (30.9)	5,094 (39.2)	92,117 (41.1)
Clinical characteristics							
Years with dialysis treatment							
<1	27,374 (5.0)	16,674 (4.5)	821 (4.5)	781 (6.1)	133 (5.7)	682 (5.2)	12,634 (5.6)
1-2	184,622 (33.5)	115,819 (31.4)	5,710 (31.4)	4,886 (38.3)	813 (34.8)	4,272 (32.9)	76,178 (34.0)
3-4	135,029 (24.5)	86,350 (23.4)	4,331 (23.8)	2,947 (23.1)	550 (23.5)	3,139 (24.1)	52,608 (23.5)
5-9	152,341 (27.6)	103,379 (28.0)	5,170 (28.4)	2,868 (22.5)	610 (26.1)	3,673 (28.3)	59,542 (26.6)
≥10	52,445 (9.5)	46,322 (12.6)	2,179 (12.0)	1,279 (10.0)	233 (10.0)	1,230 (9.5)	22,940 (10.2)
Dialysis facility characteristics							
Hospital based	34,929 (6.3)	12,713 (3.5)	942 (5.2)	1,088 (8.5)	185 (7.9)	2,930 (22.6)	17,765 (7.9)
Nonprofit	78,557 (14.2)	39,549 (10.7)	2,774 (15.2)	3,137 (24.6)	486 (20.8)	4,064 (31.3)	36,519 (16.3)
No of stations							
0-19	220,923 (40.0)	154,334 (41.9)	7,245 (39.8)	5,038 (39.5)	1,075 (46.0)	5,072 (39.0)	87,696 (39.2)
20-29	236,117 (42.8)	157,667 (42.8)	7,282 (40.0)	5,716 (44.8)	865 (37.0)	5,686 (43.8)	95,163 (42.5)
≥30	94,771 (17.2)	56,543 (15.3)	3,684 (20.2)	2,007 (15.7)	399 (17.1)	2,238 (17.2)	41,043 (18.3)
Health care utilization							
No. of days hospitalized in last month							
0	484,617 (87.8)	327,283 (88.8)	15,858 (87.1)	11,403 (89.4)	1,997 (85.4)	10,761 (82.8)	181,384 (81.0)
1-6	38,315 (6.9)	23,220 (6.3)	1,240 (6.8)	835 (6.5)	179 (7.7)	1,122 (8.6)	20,836 (9.3)
≥7	28,879 (5.2)	18,041 (4.9)	1,113 (6.1)	523 (4.1)	163 (7.0)	1,113 (8.6)	21,682 (9.7)
Skilled nursing facility in last month	31,237 (5.7)	18,537 (5.0)	1,171 (6.4)	491 (3.9)	146 (6.2)	1,173 (9.0)	21,723 (9.7)
Comorbid conditions							
Pneumonia	105,443 (19.1)	47,295 (12.8)	27,07 (14.9)	1,543 (12.1)	342 (14.6)	2,946 (22.7)	31,424 (14.0)
Ischemic heart disease	402,421 (72.9)	263,384 (71.5)	13,511 (74.2)	9,245 (72.4)	1,725 (73.8)	9,863 (75.9)	107,241 (47.9)
Dementia	107,126 (19.4)	67,245 (18.2)	3,893 (21.4)	2,129 (16.7)	454 (19.4)	3,163 (24.3)	37,712 (16.8)
Diabetes with chronic complications	369,677 (67.0)	273,437 (74.2)	13,896 (76.3)	9,625 (75.4)	1,822 (77.9)	9,132 (70.3)	102,128 (45.6)
Diabetes without chronic complications	61,692 (11.2)	18,607 (5.0)	820 (4.5)	450 (3.5)	92 (3.9)	1,268 (9.8)	13,069 (5.8)
Heart failure	396,772 (71.9)	267,586 (72.6)	13,463 (73.9)	9,194 (72.0)	1,709 (73.1)	9,764 (75.1)	108,506 (48.5)
Hypertension (uncomplicated or complicated)	545,500 (98.9)	365,540 (99.2)	18,069 (99.2)	12,680 (99.4)	2,329 (99.6)	12,896 (99.2)	141,497 (63.2)
Liver disease	108,553 (19.7)	67,444 (18.3)	3,715 (20.4)	2,258 (17.7)	417 (17.8)	2,631 (20.2)	32,723 (14.6)
Frailty markers							
Stroke or brain injury	206,879 (37.5)	155,476 (42.2)	8,178 (44.9)	5,138 (40.3)	1,064 (45.5)	5,616 (43.2)	66,873 (29.9)
Preventive health services							
Cancer screening	17,437 (3.2)	14,780 (4.0)	711 (3.9)	591 (4.6)	111 (4.8)	403 (3.1)	4,145 (1.9)
Hemoglobin A_1c_ test	300,945 (54.5)	191,014 (51.8)	10,370 (56.9)	6,850 (53.7)	1,221 (52.2)	6,550 (50.4)	74,009 (33.0)
Diabetic eye examination	179,736 (32.6)	114,364 (31.0)	5,978 (32.8)	4,448 (34.9)	815 (34.8)	4,287 (33.0)	42,593 (19.0)

Abbreviations: aIIV, adjuvanted inactivated influenza vaccine; ccIIV, cell culture-based inactivated influenza vaccine; hdIIV, high-dose inactivated influenza vaccine; RIV, recombinant influenza vaccine; SD, standard deviation; sdeIIV, standard dose, egg-based inactivated influenza vaccine.

a Results are expressed as n (%) unless otherwise indicated.

b Table S3 presents the definitions and timing of assessment for all patient characteristics.

We documented dramatic increases in uptake of newly licensed influenza vaccines among patients with kidney failure between the 2010/2011 and 2021/2022 influenza seasons, possibly reflecting changes in the formularies of large commercial dialysis providers because of increasing data of improved effectiveness of these vaccines in all persons ≥65 years. Our findings in the dialysis population generally reflect patterns in the general population. In our study, the proportion of patients ≥65 years with kidney failure in the United States who received influenza vaccination increased from 76.0% to 85.9% from 2010/2011 (24.0%) to 2020/2021, which is similar but higher than estimates in the general population (2011-2013: 59.1%; 2021-2023: 67.5%).^7^ Similar to our findings in the dialysis population, the Medicare-insured general population experienced dramatic increases over time in use of newly licensed vaccines.^4^^,^^8^ Notably, our results indicate that the dialysis population has higher uptake of hdIIV but lower uptake of other newly licensed vaccines than the Medicare population. For example, a study of 12.7 million Medicare-insured influenza vaccine recipients during the 2019/2020 influenza season reported the following distribution: 69.0% hdIIV, 24.7% aIIV, 15.0% sdeIIV, 7.9% ccIIV, and 6.3% RIV.^8^

Our study had several limitations. First, patients vaccinated in 2022 were misclassified as unvaccinated in the 2021/2022 season because of end of data (although <1% of vaccinations occurred after December 31 in each prior season). Additionally, some vaccinations may have been missed because vaccinations were identified using Current Procedural Terminology (CPT), Healthcare Common Procedure Coding System (HCPCS), International Classification of Diseases, Ninth Revision, Clinical Modification (ICD-9-CM), and International Classification of Diseases, Tenth Revision, Procedure Coding System (ICD-10-PCS) codes for insurance reimbursement purposes but not national drug codes billed by retail pharmacies; we expect this to be rare because patients with kidney failure receive in-center dialysis typically thrice weekly and receive reimbursement for influenza vaccines via Medicare Part B. Lastly, some important predictors of vaccine receipt (eg, health-seeking behaviors, personal beliefs) may not be observable in billing claims.

Our observation of increasing utilization of newly licensed influenza vaccine formulations in the dialysis population highlights the need for an updated evaluation of the comparative effectiveness and safety of influenza vaccines in this high-risk population.^9^^,^^10^
