# Supplementary material for: Uptake of Newly Licensed Influenza Vaccine Formulations Among Patients Receiving Chronic Hemodialysis During the 2010/2011 to 2021/2022 Influenza Seasons
Source: Kidney Med. 2026 Jan 6;8(3):101243. doi: 10.1016/j.xkme.2026.101243 (PMC12887787; doi:10.1016/j.xkme.2026.101243)
Supplement: Supplementary File (PDF) — Tables S1-S5 [file mmc1.pdf]

Table S1. Start and end dates of yearly influenza seasons, 2010/11 to 2021/22.

Influenza Season	Beginning date of vaccination assessment period[a]	Beginning date of influenza season[b]	End date of influenza season[c]	End date of vaccination assessment period[a]
2010-2011	August 1, 2010	November 24, 2010	April 6, 2011	April 6, 2011
2011-2012	August 1, 2011	February 1, 2012	June 6, 2012	June 6, 2012
2012-2013	August 1, 2012	November 14, 2012	April 17, 2013	April 17, 2013
2013-2014	August 1, 2013	November 27, 2013	May 21, 2014	May 21, 2014
2014-2015	August 1, 2014	November 12, 2014	April 8, 2015	April 8, 2015
2015-2016	August 1, 2015	February 10, 2016	May 4, 2016	May 4, 2016
2016-2017	August 1, 2016	December 21, 2016	April 19, 2017	April 19, 2017
2017-2018	August 1, 2017	December 6, 2017	April 18, 2018	April 18, 2018
2018-2019	August 1, 2018	December 12, 2018	April 17, 2019	April 17, 2019
2019-2020	August 1, 2019	November 27, 2019	March 18, 2020	March 18, 2020
2020-2021	August 1, 2020	N/A[d]	N/A[d]	May 31, 2021
2021-2022	August 1, 2021	N/A[d]	N/A[d]	December 31, 2021[e]

Data from the Centers for Disease Control "FLUVIEW" dashboard.[1] Centers for Disease Control. National, Regional, and State Level Outpatient Illness and Viral Surveillance. June 10, 2025. Accessed June 10, 2025. https://gis.cdc.gov/grasp/fluview/fluportaldashboard.html

[a] For each individual, we identified the first influenza vaccination received between August 1 and the end of influenza season (or 31 December 2021, the end of available data).

[b] Midpoint of the first week when >10% of national culture isolates were influenza positive for influenza, as defined by the Centers for Disease Control and Prevention.

[c] Midpoint of the first week when <10% of national culture isolates were influenza positive for influenza, as defined by the Centers for Disease Control and Prevention.

[d] No influenza season observed due to low virus circulation

[e] December 31, 2021 was the last date of available USRDS claims

Table S2. Codes to identify influenza vaccination formulations.[a]

Formulation	Code type	Codes
sdeIIV	CPT	90630, 90654, 90656, 90658, 90660, 90672, 90686, 90688
	HCPCS	Q2034-Q2038
hdIIV	CPT	90662
ccIIV	CPT	90661, 90674, 90756
aIIV	CPT	90653, 90694
RIV	CPT	90673, 90682
Unknown	HCPCS	G0008, Q2039
	ICD-9-CM procedure	99.52
	ICD-10-PCS	3E01340, 3E02340

Abbreviations: aIIV, adjuvanted inactivated influenza vaccine; ccIIV cell culture-based inactivated influenza vaccine; CPT, Current Procedural Terminology; HCPCS, Healthcare Common Procedure Coding System; hdIIV, high dose inactivated influenza vaccine; ICD-9-CM, International Classification of Diseases, Ninth Revision, Clinical Modification; ICD-10-PCS, International Classification of Diseases, Tenth Revision, Procedure Coding System; RIV, recombinant influenza vaccine; sdeIIV, standard dose, egg-based inactivated influenza vaccine.

[a] Within each season, influenza vaccines were identified with billing claims as specified above.

Table S3. Definitions of patient characteristics.

Patient characteristic	Definition	Timing [a]
Demographic		
Age	Age (years); modeled using restricted cubic splines	Index date
Sex	Male or female	Index date
Race	Black, white, or other	Index date
Region	Northeast, Midwest, South, or West	Index date
Residence in metropolitan statistical area	Patient zip code within metropolitan statistical area[b]	Index date
Medicaid eligibility	Dual-eligible for Medicaid during baseline period	Index date
Week of vaccination	Weeks since August 1 and vaccination; modeled using restricted cubic splines	Index date
Clinical characteristics		
Cause of kidney failure	Diabetes, hypertension, glomerulonephritis, or other	Index date
Years on dialysis	Years of kidney failure services; modeled using restricted cubic splines	Index date
Concomitant vaccination	Pneumococcal vaccine, hepatitis B vaccine, shingles vaccine, or COVID-19 vaccine	Index date
Healthcare utilization		
Hospitalized in last month	0 days, 1-6 days, or >= 7 days	30 days before index date
Skilled nursing facility in last month	At least one day in SNF in last month	30 days before index date
Comorbidities		
Deficiency anemias	ICD-9-CM diagnosis<br>280.1-281.9, 285.21-285.29, 285.9<br>ICD-10-CM<br>D50.1-D53.9, D63.0-D63.8, D64.9, O99.011-O99.019	Baseline
Chronic blood loss	ICD-9-CM diagnosis<br>280.0, 648.20-648.24<br>ICD-10-CM<br>D50.0	Baseline
Autoimmune arthritis	ICD-9-CM diagnosis<br>710.1-710.9, 711.10-711.39, 714.0-714.9, 716.80-716.89, 725<br>ICD-10-CM<br>M05.00-M06.1, M06.30-M08.0A, M08.20-M08.9A, M12.00-M12.09, M33.00-M35.04,	All available lookback

	M35.09, M35.1-M35.3, M35.5, M35.8-M35.9, M36.8	
Other arthritis	ICD-9-CM diagnosis<br>711.00-711.09, 711.40-712.99, 716.50-716.68, 716.90-716.99, 718.00-719.19, 719.40-719.59, 719.90-719.99<br>ICD-10-CM	All available lookback
Lupus	ICD-9-CM diagnosis<br>710.0<br>ICD-10-CM<br>M32.0-M32.9	All available lookback
Other autoimmune disorders	ICD-9-CM diagnosis<br>099.3, 136.1, 446.0-446.7, 447.5, 696.0, 701.0, 713.1, 713.6-713.8, 720.0-720.9<br>ICD-10-CM<br>A18.01-A18.02, A39.84, A54.41-A54.42, L40.50-L40.51, L40.54-L40.59, L90.0, L94.0-L94.1, L94.3, M06.20-M06.29, M08.1, M30.0-M31.9, M35.05-M35.08, M35.0A-M35.0C, M35.6, M36.0-M36.1, M45.0-M45.AB	All available lookback
Viral hepatitis	ICD-9-CM diagnosis<br>070.0-070.9, V02.60-V02.69<br>ICD-10-CM<br>B15.0-B19.9, Z22.50-Z22.59	All available lookback
HIV/AIDS	ICD-9-CM diagnosis<br>042, V08<br>ICD-10-CM<br>B20, O98.711-O98.73, Z21	All available lookback
Alcohol abuse	ICD-9-CM diagnosis<br>291.0-291.3, 291.5-291.9, 303.00-303.93, 305.00-305.03, 357.5, 425.5, 535.30-535.31, 571.1<br>ICD-10-CM<br>F10.10-F10.29, F10.94-F10.980, G62.1, I42.6, K29.20-K29.21, K70.10-K70.11, O99.310-O99.315	All available lookback
Depression	ICD-9-CM diagnosis<br>293.83, 300.4, 301.12, 309.0-309.1, 311<br>ICD-10-CM<br>F06.31-F06.32, F06.34, F32.0-F32.3, F32.8-F33.3, F33.8-F33.9, F34.1	All available lookback
Obesity	ICD-9-CM diagnosis	All available lookback

	278.00-278.01, 278.03, 649.10-649.14, 793.91, V85.30-V85.45, V85.54<br>ICD-10-CM<br>E66.01-E66.2, E66.8-E66.9, O99.210-O99.215, R93.9, Z68.30-Z68.45, Z68.54	
Peripheral vascular disease	ICD-9-CM diagnosis<br>093.0-093.1, 093.89-093.9, 440.0-443.9, 444.01-445.89, 447.0-448.0, 448.9-449, 459.89-459.9, 537.82-537.83, 557.1-557.9, V43.4<br>ICD-10-CM<br>A52.00-A52.02, A52.09, I70.0-I72.9, I73.01-I75.89, I77.0-I79.8, K55.1, Z95.820-Z95.828	All available lookback
Hypothyroidism	ICD-9-CM diagnosis<br>240.9, 241.1, 243-244.9, 246.8, 780.01<br>ICD-10-CM<br>E00.0-E03.9, E89.0	All available lookback
Other thyroid disorders	ICD-9-CM diagnosis<br>240.0-242.91, 245.0-245.9, 246.2, 648.12, 648.14<br>ICD-10-CM<br>E04.0-E06.9, O90.5	All available lookback
Valvular disease	ICD-9-CM diagnosis<br>093.20-093.24, 391.1, 391.8-392.0, 394.0-397.9, 421.0, 421.9, 424.0-424.99, 746.00-746.7, 746.89, 996.02, 996.09, V42.2, V43.3<br>ICD-10-CM<br>A18.84, A32.82, A39.51, A52.03, B33.21, B37.6, I01.1, I01.8-I02.0, I05.0-I08.9, I09.1, I09.89, I33.0-I39, M32.11, Q22.0-Q23.9, T82.01XA-T82.09XS, T82.221A-T82.228S, T82.6XXA-T82.6XXS, Z95.2-Z95.4	All available lookback
Weight loss	ICD-9-CM diagnosis<br>260-263.9, 783.21-783.22, 799.4<br>ICD-10-CM<br>E40-E46, E64.0, O25.10-O25.3, R63.4, R64	Baseline
Metastatic cancer	ICD-9-CM diagnosis<br>196.0-199.1, 209.70-209.79, 789.51<br>ICD-10-CM<br>C77.0-C79.9, C7B.00-C80.0	All available lookback
Solid tumor without metastasis	ICD-9-CM diagnosis<br>140.0-172.9, 173.00, 173.09, 173.10, 173.19, 173.20, 173.29, 173.30, 173.39, 173.40, 173.49, 173.50, 173.59, 173.60, 173.69, 173.70, 173.79,	All available lookback

	173.80, 173.89, 173.90, 173.99-195.8, 209.00-209.36, 238.75, 258.01-258.03<br>ICD-10-CM<br>C00.0-C43.9, C45.0-C49.A9, C50.011-C76.8, C7A.00-C7A.8, D46.9, E31.21-E31.23	
Leukemia	ICD-9-CM diagnosis<br>202.40-202.48, 203.10-203.12, 204.00-207.02, 207.20-208.92, 238.79<br>ICD-10-CM<br>C90.10-C90.12, C91.00-C95.92	All available lookback
Lymphoma	ICD-9-CM diagnosis<br>200.00-202.38, 202.50-203.02, 203.80-203.82, 238.6, 273.3<br>ICD-10-CM<br>C81.00-C90.02, C90.20-C90.32, C96.0-C96.4, C96.9-C96.Z, D47.Z9	All available lookback
Coagulopathy	ICD-9-CM diagnosis<br>284.09, 284.11-284.2, 284.89-284.9, 286.0-286.9, 287.1, 287.30-287.5, 289.84, 649.30-649.34<br>ICD-10-CM<br>D61.09-D61.9, D65-D68.4, D68.8-D68.9, D69.1, D69.3-D69.9, D75.82-D75.829, D75.84, O99.111-O99.13	All available lookback
Chronic pulmonary disease	ICD-9-CM diagnosis<br>490-505, 506.4, 508.1, 508.8<br>ICD-10-CM<br>J41.0-J67.9, J68.4, J70.1, J70.3	All available lookback
Diabetes without chronic complications	ICD-9-CM diagnosis<br>249.00-249.31, 250.00-250.33, 648.00-648.04<br>ICD-10-CM<br>E08.00-E08.11, E08.9-E09.11, E09.9-E10.11, E10.9-E11.11, E11.9-E13.11, E13.9, O24.011-O24.93	All available lookback
Diabetes with chronic complications	ICD-9-CM diagnosis<br>249.40-249.91, 250.40-250.93, 775.1<br>ICD-10-CM<br>E08.21-E08.8, E09.21-E09.8, E10.21-E10.8, E11.21-E11.8, E13.21-E13.8	All available lookback
Dementia	ICD-9-CM diagnosis<br>290.0-290.9, 294.10-294.21, 330.8-331.2, 331.6-331.7, 331.82, 331.89-331.9<br>ICD-10-CM	All available lookback

	F01.50-F03.C4, F06.70-F06.71, G30.0-G31.83, G31.85-G31.9	
Gastrointestinal bleeding	ICD-9-CM diagnosis<br>531.00-531.01, 531.20-531.21, 531.40-531.41, 531.60-531.61, 532.00-532.01, 532.20-532.21, 532.40-532.41, 532.60-532.61, 533.00-533.01, 533.20-533.21, 533.40-533.41, 533.60-533.61, 562.02-562.03, 562.12-562.13, 569.3, 569.85-569.86, 578.1-578.9, 792.1<br>ICD-10-CM<br>K25.0, K25.2, K25.4, K25.6, K26.0, K26.2, K26.4, K26.6, K27.0, K27.2, K27.4, K27.6, K55.21, K56.60-K56.609, K56.699, K57.01, K57.11, K57.13, K57.21, K57.31, K57.33, K57.41, K57.51, K57.53, K57.81, K57.91, K57.93, K62.5, K63.81, K92.1-K92.2, R19.5	Baseline
Heart failure	ICD-9-CM diagnosis<br>398.91, 402.01, 402.11, 402.91, 404.01, 404.03, 404.11, 404.13, 404.91, 404.93, 428.0-428.9, 429.83, 785.51, V43.21-V43.22<br>ICD-10-CM<br>I09.81, I11.0, I13.0, I13.2, I50.1-I50.9, I51.81, I97.130-I97.131, O29.121-O29.129, R57.0, Z95.811-Z95.812	All available lookback
Hypertension	ICD-9-CM diagnosis<br>362.11, 401.0-405.99, 437.2, 642.00-642.24, 642.70-642.94<br>ICD-10-CM H35.031-H35.039, I10-I16.9, I67.4, O10.011-O11.9, O16.1-O16.9	All available lookback
Ischemic heart disease	ICD-9-CM diagnosis<br>411.0-411.89, 413.0-414.9<br>ICD-10-CM<br>I20.0-I20.9, I23.0-I25.119, I25.3-I25.9	All available lookback
Liver disorders	ICD-9-CM diagnosis<br>070.20, 070.22-070.30, 070.32-070.33, 070.44, 070.54-070.9, 078.5, 091.62, 095.2-095.3, 130.5, 456.0-456.21, 456.8, 570-571.9, 572.1-573.9, V42.7<br>ICD-10-CM<br>A51.45, A52.74, B18.0-B19.9, B25.1, B58.1, I85.00-I85.11, I86.4, K70.0-K70.9, K71.3-K71.8, K72.10-K74.69, K75.1-K77, K91.82, Z94.4	All available lookback
Myocardial infarction	ICD-9-CM diagnosis	All available lookback

	410.00-410.92, 412<br>ICD-10-CM<br>I21.01-I22.9, I25.2	
Neurological disorders affecting movement	ICD-9-CM diagnosis<br>325, 331.7, 332.0-333.0, 333.4-333.79, 333.84, 333.90-335.9, 336.2, 349.89, 357.3<br>ICD-10-CM<br>G08, G10-G13.8, G20-G23.9, G24.09-G24.2, G24.8, G25.4-G25.5, G25.70-G26, G32.0-G32.89, G80.3	All available lookback
Other neurological disorders	ICD-9-CM diagnosis<br>290.12, 290.20, 290.3, 293.0-293.1, 323.9, 330.0-330.8, 331.3-331.5, 331.7-331.81, 338.0, 340-341.9, 347.00-348.0, 348.30-348.5, 348.81-348.9, 649.40-649.44, 768.7-768.73<br>ICD-10-CM<br>E75.00-E75.19, E75.23, E75.25-E75.29, E75.4, F05, F84.2, G35-G36.0, G36.8-G37.9, G47.411-G47.429, G89.0, G91.0-G91.9, G93.0, G93.40-G94, K76.82, O99.350-O99.355, P91.60-P91.63	All available lookback
Seizures and epilepsy	ICD-9-CM diagnosis<br>345.00-345.91, 780.33-780.39<br>ICD-10-CM<br>G40.001-G40.B19, R56.1-R56.9	All available lookback
Peptic ulcer disease	ICD-9-CM diagnosis<br>531.41, 531.51, 531.61-531.71, 531.91, 532.41, 532.51, 532.61-532.71, 532.91, 533.41, 533.51, 533.61-533.71, 533.91, 534.41, 534.51, 534.61-534.71, 534.91<br>ICD-10-CM<br>K25.0-K28.9	All available lookback
Pneumonia	ICD-9-CM diagnosis<br>002.0, 003.22, 006.4, 020.3-020.5, 021.2, 022.1, 031.0, 033.0-033.9, 038.0, 038.2, 039.1, 041.09, 041.2-041.3, 041.81, 052.1, 055.1, 056.79, 073.0, 090.0, 098.89, 112.4, 114.0, 114.5, 115.05, 115.15, 115.95, 116.0-116.1, 121.2, 122.1, 127.0, 130.4, 136.3, 466.0, 480.0-483.8, 484.3-484.5, 484.7-487.0, 488.01, 488.11, 488.81, 513.0, 514, 516.30, 516.35-516.37, 517.1, 770.0, 995.91, 997.31, V12.61<br>ICD-10-CM<br>A01.03, A02.22, A06.5, A20.2, A21.2, A22.1,	Baseline

	A31.0, A37.01, A37.11, A37.81, A37.91, A40.3, A42.0, A43.0, A48.1, A50.04, A54.84, B01.2, B05.2, B06.81, B37.1, B38.0, B38.2, B39.0, B39.2, B40.0, B40.2, B41.0, B58.3, B59, B66.4, B67.1, B77.81, B95.3, B96.0-B96.1, J09.X1, J10.00-J10.08, J11.00-J11.08, J12.0-J20.0, J84.111, J84.116-J84.117, J84.178-J84.2, J85.1, J95.851, P23.0-P23.9, Z87.01	
Psychoses	ICD-9-CM diagnosis<br>292.11-292.12, 292.89, 293.81-293.83, 294.8, 295.00-298.9, 299.10-299.11, 301.10, 301.13, 301.22<br>ICD-10-CM<br>F06.0-F06.30, F06.33, F11.150-F11.159, F11.250-F11.259, F11.950-F11.959, F12.150-F12.159, F12.250-F12.259, F12.950-F12.959, F13.150-F13.159, F13.250-F13.259, F13.950-F13.959, F14.150-F14.159, F14.250-F14.259, F14.950-F14.959, F15.150-F15.159, F15.250-F15.259, F15.950-F15.959, F16.150-F16.159, F16.250-F16.259, F16.950-F16.959, F18.150-F18.159, F18.250-F18.259, F18.950-F18.959, F19.150-F19.159, F19.250-F19.259, F19.950-F19.959, F20.0-F31.9, F32.4-F32.5, F33.40-F33.42, F34.0, F34.8-F39, F44.89, F84.3	All available lookback
Pulmonary circulation disease	ICD-9-CM diagnosis<br>415.11-417.9<br>ICD-10-CM<br>I27.0-I28.9	All available lookback
Sepsis	ICD-9-CM diagnosis<br>038.0-038.9, 995.90-995.92<br>ICD-10-CM<br>A02.1, A22.7, A26.7, A32.7, A40.0-A41.9, A42.7, A54.86, B37.7, R65.10, R65.20-R65.21	Baseline
Substance abuse	ICD-9-CM diagnosis<br>292.0, 292.82-292.9, 304.00-304.93, 305.20-305.93, 648.30-648.34<br>ICD-10-CM<br>F11.10-F11.29, F12.10-F12.29, F13.10-F13.29, F14.10-F14.29, F15.10-F15.29, F16.10-F16.29, F18.10-F18.29, F19.10-F19.29, O99.320-O99.325	All available lookback
Transfusion	CPT/HCPCS	Baseline

	P9010, P9011, P9016, P9021, P9022, P9038, P9039, P9040, 36430<br>ICD-9-CM procedure<br>99.03-99.04<br>ICD-10-PCS<br>30230H1, 30230N1, 30230P1, 30233H1, 30233N1, 30233P1, 30240H1, 30240N1, 30240P1, 30243H1, 30243N1, 30243P1, 30250H1, 30250N1, 30250P1, 30253H1, 30253N1, 30253P1, 30260H1, 30260N1, 30260P1, 30263H1, 30263N1, 30263P1	
Vascular infection	ICD-9-CM diagnosis<br>996.62<br>ICD-10-CM<br>T82.7XXA	Baseline
Hypotension or shock	ICD-9-CM diagnosis<br>458.0-458.9, 785.50-785.59, 958.4, 998.0-998.09<br>ICD-10-CM<br>I95.0-I95.9, R57.0-R57.9, R65.20-R65.21, T79.4XXA-T79.4XXS, T81.10XA-T81.19XS	Baseline
Lipid abnormality	ICD-9-CM diagnosis<br>272.0-272.9<br>ICD-10-CM<br>E71.30, E75.21-E75.22, E75.240-E75.249, E75.3, E75.5-E75.6, E77.0, E78.0-E78.70, E78.79-E78.9, E88.1, E88.89	All available lookback
Paralysis	ICD-9-CM diagnosis<br>342.00-342.92, 344.00-344.9, 438.20-438.53, 781.4<br>ICD-10-CM<br>G81.00-G82.54, G83.10-G83.34, G83.5, G83.89-G83.9, I69.031-I69.069, I69.131-I69.169, I69.231-I69.269, I69.331-I69.369, I69.831-I69.869, I69.931-I69.969, R29.5	All available lookback
Stroke or brain injury	ICD-9-CM diagnosis<br>348.0-348.9, 349.82, 430-432.9, 433.01, 433.11, 433.21, 433.31, 433.91, 434.01, 434.11, 434.91, 436, 852.00-854.19<br>ICD-10-CM<br>G92-G92.9, G93.1, G93.40-G93.6, G93.89-G93.9, I60.00-I63.039, I63.10-I63.139, I63.20-I63.9, I67.83, I67.89, S01.90XA-S01.90XS, S06.1X0A-	All available lookback

	S06.309S, S06.340A-S06.369S, S06.4X0A-S06.9X9S	
Frailty indicators		
Ambulance / life support	HCPCS<br>A0426, A0427, A0428, A0429, A0999	Baseline
Difficulty walking	ICD-9-CM diagnosis<br>438.85, 719.7, 781.2-781.3, V46.3<br>ICD-10-CM<br>I69.998, R26.0-R27.9, Z99.3	Baseline
Bladder dysfunction	ICD-9-CM diagnosis<br>596.51-596.59, 599.60-599.69, 788.20-788.39<br>ICD-10-CM<br>N13.0-N13.9, N31.0-N32.9, N36.0-N36.9, N39.3-N39.9, R32-R33.9, R39.14, R39.81	Baseline
Home hospital bed	HCPCS<br>E0250, E0251, E0255, E0256, E0260, E0261, E0265, E0266, E0270, E0290, E0291, E0292, E0293, E0294, E0295, E0296, E0297, E0301, E0302, E0303, E0304, E0316	Baseline
Wheelchair	HCPCS<br>E1050, E1060, E1070, E1083, E1084, E1085, E1086, E1087, E1088, E1089, E1090, E1091, E1092, E1093, E1100, E1110, E1140, E1150, E1160, E1161, E1170, K0001, K0002, K0003, K0004, K0005, K0006, K0007, K0008, K0009	All available lookback
Podiatric care	ICD-9-CM diagnosis<br>681.10-681.11, 700, 703.0-703.9<br>ICD-10-CM<br>L02.611-L02.619, L03.031-L03.049, L60.0-L62, L84	Baseline
Rehabilitation services	CPT<br>92507, 97012, 97110, 97112, 97113, 97116, 97161, 97162, 97164, 97530, 97535<br>ICD-9-CM diagnosis<br>V57.1-V57.21, V57.3, V57.81-V57.9<br>ICD-10-CM<br>Z51.89	Baseline
Skin ulcer (decubitus)	ICD-9-CM diagnosis<br>707.00-707.9<br>ICD-10-CM<br>L89.000-L89.96, L97.101-L97.929, L98.411-L98.499	Baseline

Home oxygen	HCPCS<br>E0431, E0433, E0434, E0435, E0439, E0441, E0442, E0443, E1390, E1391, E1392	Baseline
Weakness	ICD-9-CM diagnosis<br>728.2-728.3, 728.87, 799.3, V49.84<br>ICD-10-CM<br>M62.50-M62.81, M62.84-M62.89, R53.81, R54, Z74.01-Z74.09	Baseline
Vertigo	ICD-9-CM diagnosis<br>386.00-386.9, 438.85, 780.4<br>ICD-10-CM<br>H81.01-H83.2X9, R42	Baseline
Preventive health services		
HbA_{1c}	CPT<br>83036, 83037	Baseline
Diabetic eye exam	CPT/HCPCS<br>67028, 67030, 67031, 67036, 67038, 67039, 67041, 67042, 67043, 67101, 67105, 67107, 67108, 67110, 67112, 67113, 67121, 67141, 67145, 67208, 67210, 67218, 67221, 67227, 67228, 67228, 92002, 92004, 92012, 92014, 92018, 92019, 92201, 92202, 92225, 92226, 92230, 92235, 92240, 92250, 92260, S0620, S0621, S0625, S3000	Baseline
Lipid test	CPT<br>80061	Baseline
Cancer screening	ICD-9-CM diagnosis<br>V76.0-V76.9<br>ICD-10-CM<br>Z12.0-Z12.9	Baseline
Hepatitis B vaccine	CPT/HCPCS<br>90636, 90723, 90731, 90739, 90740, 90743, 90744, 90746, 90747, 90748, 90859, G0010	Baseline
Pneumococcal vaccine	CPT/HCPCS<br>90669, 90670, 90671, 90677, 90732, G0009	Baseline
Shingles vaccine	CPT<br>90736, 90750	Baseline
COVID-19 vaccine	CPT<br>91300, 91301, 91302, 91303, 91304, 91305, 91306	Baseline

Abbreviations: COPD, chronic obstructive pulmonary disease.

[a] The index date was anchored on the date of influenza vaccination, which was required to be administered prior to the start date of the influenza season. For unvaccinated patients, the index date was randomly assigned based on the distribution of vaccination dates among vaccinated patients. Baseline variables were ascertained during the 6-month baseline period prior to the index date. All available lookback used claims starting the day before index and extending as far back as January 1, 2008.
[b] Residence in a metropolitan statistical area determined by mapping patient ZIP code to metropolitan statistical area using the Department of Housing and Urban Development's United States Postal Service Zip code crosswalk files (https://www.huduser.gov/portal/datasets/usps_crosswalk.html).

Table S4. Additional demographic and clinical characteristics of patients with kidney failure receiving chronic hemodialysis during the 2010/11 to 2021/22 influenza seasons in the U.S., by influenza vaccine formulation

**Characteristic**	**sdeIIV N=551,811 46.3%**	**hdIIV N=368,544 31.0%**	**ccIIV N=18,211 1.5%**	**aIIV N=12,761 1.1%**	**RIV N=2,339 0.2%**	**Unknown N=12,996 1.1%**	**Unvaccinated N=223,902 18.8%**
Demographics							
Influenza season							
2010/11	67,497 (12.2)	557 (0.1)	0 (0.0)	0 (0.0)	0 (0.0)	543 (4.2)	21,605 (9.7)
2011/12	68,264 (12.4)	1422 (0.4)	0 (0.0)	0 (0.0)	0 (0.0)	2332 (17.9)	21,485 (9.6)
2012/13	70,659 (12.8)	1645 (0.4)	<11*	0 (0.0)	0 (0.0)	1279 (9.8)	19,754 (8.8)
2013/14	73,386 (13.3)	2341 (0.6)	<11*	<11*	0 (0.0)	2035 (15.7)	20,234 (9.0)
2014/15	76,891 (13.9)	3347 (0.9)	564 (3.1)	<11*	18 (0.8)	1123 (8.6)	18,176 (8.1)
2015/16	77,087 (14.0)	4212 (1.1)	367 (2.0)	<11*	18 (0.8)	988 (7.6)	18,099 (8.1)
2016/17	55,806 (10.1)	25,152 (6.8)	306 (1.7)	178 (1.4)	18 (0.8)	979 (7.5)	22,160 (9.9)
2017/18	21,439 (3.9)	61,340 (16.6)	1924 (10.6)	793 (6.2)	183 (7.8)	1873 (14.4)	17,915 (8.0)
2018/19	15,975 (2.9)	68,248 (18.5)	4007 (22.0)	1128 (8.8)	381 (16.3)	488 (3.8)	16,969 (7.6)
2019/20	11,318 (2.0)	71,528 (19.4)	4235 (23.3)	2128 (16.7)	793 (33.9)	524 (4.0)	17,319 (7.7)
2020/21	7098 (1.3)	74,459 (20.2)	4138 (22.7)	3439 (27.0)	531 (22.7)	494 (3.8)	14,812 (6.6)
2021/22	6391 (1.2)	54,293 (14.7)	2662 (14.6)	5087 (39.9)	397 (17.0)	338 (2.6)	15,374 (6.9)
Weeks between August 1 and index, median (IQR)[a]	5 (5, 9)	5 (5, 9)	9 (5, 9)	8 (6, 10)	9 (8, 11)	8 (5, 9)	8 (5, 9)

Clinical characteristics							
Cause of kidney failure							
Diabetes	267,001 (48.4)	176,709 (48.0)	8784 (48.2)	5948 (46.6)	1152 (49.2)	6371 (49.0)	105,255 (47.0)
Hypertension	175,664 (31.8)	118,999 (32.3)	5879 (32.3)	4118 (32.3)	728 (31.1)	4056 (31.2)	75,363 (33.7)
Glomerulonephritis	35,388 (6.4)	24,445 (6.6)	1172 (6.4)	852 (6.7)	158 (6.8)	813 (6.3)	13,035 (5.8)
Other	73,758 (13.4)	48,391 (13.1)	2376 (13.1)	1843 (14.4)	301 (12.9)	1756 (13.5)	30,249 (13.5)
Concomitant vaccination	1759 (0.3)	2182 (0.6)	114 (0.6)	391 (3.1)	56 (2.4)	90 (0.7)	15 (0.0)
Comorbidities							
Gastrointestinal bleeding	44,978 (8.2)	32,455 (8.8)	1776 (9.8)	1204 (9.4)	241 (10.3)	1357 (10.4)	15,849 (7.1)
Sepsis	47,514 (8.6)	31,194 (8.5)	1850 (10.2)	1013 (7.9)	260 (11.1)	1593 (12.3)	19,752 (8.8)
Vascular infection	17,392 (3.1)	7610 (2.1)	466 (2.6)	248 (1.9)	56 (2.4)	497 (3.8)	5629 (2.5)
Autoimmune arthritis	42,498 (7.7)	27,620 (7.5)	1387 (7.6)	1002 (7.8)	167 (7.1)	1018 (7.8)	11,672 (5.2)
Lupus	6367 (1.1)	4975 (1.4)	246 (1.4)	180 (1.4)	29 (1.2)	152 (1.2)	1881 (0.8)
Other arthritis	313,406 (56.8)	242,018 (65.7)	12,351 (67.8)	8522 (66.8)	1566 (67.0)	7854 (60.4)	89,195 (39.8)
Other autoimmune disorders	22,508 (4.1)	14,287 (3.9)	665 (3.6)	494 (3.9)	85 (3.6)	560 (4.3)	5733 (2.6)
Viral hepatitis	32,052 (5.8)	24,603 (6.7)	1508 (8.3)	870 (6.8)	165 (7.0)	850 (6.5)	11,528 (5.2)
Myocardial infarction	184,675 (33.5)	135,687 (36.8)	7087 (38.9)	4727 (37.0)	947 (40.5)	4749 (36.5)	55,100 (24.6)
Acquired immune deficiency syndrome	3751 (0.7)	3081 (0.8)	211 (1.2)	123 (1.0)	20 (0.9)	138 (1.1)	1481 (0.7)
Alcohol abuse	17,608 (3.2)	14,559 (4.0)	842 (4.6)	500 (3.9)	86 (3.7)	501 (3.9)	6264 (2.8)

Deficiency anemias	260,486 (47.2)	179,426 (48.7)	10,594 (58.2)	6582 (51.6)	1345 (57.5)	7127 (54.8)	80,810 (36.1)
Chronic blood loss anemia	15,025 (2.7)	11,336 (3.1)	709 (3.9)	394 (3.1)	81 (3.5)	480 (3.7)	5743 (2.6)
Chronic pulmonary disease	277,796 (50.3)	174,053 (47.2)	8875 (48.7)	5788 (45.4)	1151 (49.2)	6808 (52.4)	74,320 (33.2)
Depression	159,810 (29.0)	125,877 (34.2)	6762 (37.1)	4445 (34.8)	848 (36.2)	4509 (34.7)	51,431 (23.0)
Drug abuse	13,247 (2.4)	15,538 (4.2)	851 (4.7)	518 (4.1)	106 (4.5)	422 (3.2)	5660 (2.5)
Leukemia	7456 (1.4)	4496 (1.2)	255 (1.4)	207 (1.6)	35 (1.5)	194 (1.5)	1968 (0.9)
Lymphoma	16,214 (2.9)	11,888 (3.2)	618 (3.4)	537 (4.2)	79 (3.4)	403 (3.1)	4620 (2.1)
Metastatic cancer	18,616 (3.4)	12,153 (3.3)	640 (3.5)	466 (3.6)	81 (3.5)	474 (3.6)	5548 (2.5)
Neurological disorders affecting movement	48,552 (8.8)	36,438 (9.9)	1733 (9.5)	1248 (9.8)	285 (12.2)	1293 (9.9)	13,557 (6.0)
Other neurological disorders	139,011 (25.2)	114,406 (31.0)	6020 (33.1)	3813 (29.9)	796 (34.0)	4088 (31.5)	49,621 (22.2)
Obesity	159,152 (28.8)	155,080 (42.1)	7527 (41.3)	5424 (42.5)	991 (42.4)	3944 (30.4)	48,311 (21.6)
Peripheral vascular disease	404,513 (73.3)	267,263 (72.5)	13,696 (75.2)	9385 (73.5)	1695 (72.5)	9955 (76.6)	108,984 (48.7)
Psychoses	123,558 (22.4)	52,538 (14.3)	2898 (15.9)	1595 (12.5)	365 (15.6)	3293 (25.3)	36,193 (16.2)
Pulmonary circulation disease	151,871 (27.5)	114,405 (31.0)	5636 (31.0)	3921 (30.7)	729 (31.2)	3953 (30.4)	44,780 (20.0)
Seizures and epilepsy	64,558 (11.7)	44,519 (12.1)	2568 (14.1)	1406 (11.0)	323 (13.8)	1880 (14.5)	22,197 (9.9)
Hypothyroidism	171,439 (31.1)	115,271 (31.3)	5636 (31.0)	4119 (32.3)	791 (33.8)	4208 (32.4)	45,530 (20.3)
Other thyroid disorders	47,070 (8.5)	36,860 (10.0)	1917 (10.5)	1363 (10.7)	252 (10.8)	1166 (9.0)	13,869 (6.2)

Solid tumor without metastasis (excluding non-melanoma skin cancers)	102,897 (18.6)	70,186 (19.0)	3438 (18.9)	2696 (21.1)	474 (20.3)	2464 (19.0)	26,481 (11.8)
Peptic ulcer disease	13,619 (2.5)	34,302 (9.3)	1920 (10.5)	1433 (11.2)	270 (11.5)	534 (4.1)	8424 (3.8)
Valvular disease	321,527 (58.3)	225,631 (61.2)	11,270 (61.9)	7798 (61.1)	1412 (60.4)	7979 (61.4)	88,321 (39.5)
Weight loss	167,895 (30.4)	116,968 (31.7)	5968 (32.8)	3895 (30.5)	747 (31.9)	4326 (33.3)	53,460 (23.9)
Transfusion	39,894 (7.2)	17,483 (4.7)	1182 (6.5)	605 (4.7)	148 (6.3)	1251 (9.6)	13,960 (6.2)
Frailty indicators							
Bladder dysfunction	24,386 (4.4)	22,746 (6.2)	1297 (7.1)	915 (7.2)	178 (7.6)	717 (5.5)	8673 (3.9)
Hypotension or shock	76,134 (13.8)	59,940 (16.3)	3230 (17.7)	2136 (16.7)	432 (18.5)	2273 (17.5)	28,300 (12.6)
Podiatric care	77,793 (14.1)	51,732 (14.0)	2849 (15.6)	1960 (15.4)	342 (14.6)	2023 (15.6)	21,231 (9.5)
Decubitus skin ulcer	76,363 (13.8)	49,767 (13.5)	2763 (15.2)	1677 (13.1)	357 (15.3)	2332 (17.9)	26,573 (11.9)
Vertigo	37,281 (6.8)	24,541 (6.7)	1338 (7.3)	919 (7.2)	182 (7.8)	937 (7.2)	10,283 (4.6)
Weakness	93,187 (16.9)	71,240 (19.3)	4051 (22.2)	2388 (18.7)	511 (21.9)	3018 (23.2)	36,902 (16.5)
Ambulance transport	189,483 (34.3)	119,601 (32.5)	6468 (35.5)	3763 (29.5)	754 (32.2)	5291 (40.7)	62,890 (28.1)
Home hospital bed	21,534 (3.9)	10,137 (2.8)	535 (2.9)	303 (2.4)	66 (2.8)	601 (4.6)	6179 (2.8)
Home oxygen	73,802 (13.4)	40,645 (11.0)	1775 (9.8)	1345 (10.5)	286 (12.2)	1771 (13.6)	17,142 (7.7)
Difficulty walking	97,860 (17.7)	75,745 (20.6)	4400 (24.2)	2699 (21.1)	541 (23.1)	3034 (23.4)	36,012 (16.1)

Lipid abnormality	463,180 (83.9)	326,169 (88.5)	16,143 (88.6)	11,645 (91.2)	2109 (90.2)	11,091 (85.3)	122,224 (54.6)
Paralysis	60,265 (10.9)	43,808 (11.9)	2513 (13.8)	1317 (10.3)	277 (11.8)	1671 (12.9)	21,411 (9.6)
Rehabilitation services	54,339 (9.8)	21,557 (5.8)	1151 (6.3)	901 (7.1)	169 (7.2)	1454 (11.2)	14,338 (6.4)
Wheelchair use	112,770 (20.4)	66,182 (18.0)	3351 (18.4)	1934 (15.2)	383 (16.4)	2820 (21.7)	31,338 (14.0)
Preventive health services							
Hepatitis B vaccine	341 (0.1)	266 (0.1)	22 (0.1)	14 (0.1)	<11*	<11*	97 (0.0)
Lipid test	131,082 (23.8)	105,866 (28.7)	8620 (47.3)	4827 (37.8)	856 (36.6)	3431 (26.4)	35,676 (15.9)
Pneumonia vaccine	6547 (1.2)	5673 (1.5)	263 (1.4)	258 (2.0)	49 (2.1)	146 (1.1)	1622 (0.7)
COVID-19 vaccine	74 (0.0)	353 (0.1)	30 (0.2)	35 (0.3)	<11*	<11*	72 (0.0)
Shingles vaccine	551 (0.1)	421 (0.1)	19 (0.1)	28 (0.2)	<11*	18 (0.1)	82 (0.0)

Abbreviations: aIIV, adjuvanted inactivated influenza vaccine; ccIIV cell culture-based inactivated influenza vaccine; hdIIV, high dose inactivated influenza vaccine; IQR, interquartile range; RIV, recombinant influenza vaccine; sdeIIV, standard dose, egg-based inactivated influenza vaccine.

[a] The timing of vaccination varied across calendar year, potentially due to emphasis on early vaccination in some years. Observed differences in timing of vaccination by formulation overall are due to the relative frequency of each formulation in different years rather than differences between formulations within individual years.
* Frequencies <11 suppressed following USRDS publication guidelines.

Table S5. Temporal trends in demographic characteristics of patients with end-stage kidney disease receiving chronic hemodialysis during the 2014/15 to 2021/22 influenza seasons in the U.S., by influenza vaccine formulation.

**Characteristic**	**2014/15**	**2015/16**	**2016/17**	**2017/18**	**2018/19**	**2019/20**	**2020/21**	**2021/22**
sdeIIV								
No. of individuals	76,891	77,087	55,806	21,439	15,975	11,318	7098	6391
Age, mean (SD)	74.6 (7.1)	74.5 (7.1)	74.5 (7.1)	74.2 (7.1)	74.0 (7.0)	74.0 (7.1)	73.8 (7.0)	74.5 (7.2)
Male	50.1	50.5	50.6	50.6	50.4	51.2	50.8	52.6
Race								
White	61.0	60.3	59.9	59.4	58.7	60.0	60.2	61.5
Black	32.1	32.4	32.3	31.8	32.5	30.4	29.9	25.6
Other	7.0	7.3	7.9	8.8	8.8	9.6	10.0	12.9
Residence in metropolitan statistical area	88.2	88.0	88.7	87.5	87.2	87.2	86.9	89.9
Region								
Northeast	17.0	17.1	16.8	17.4	18.5	16.5	16.2	16.8
South	44.5	44.8	44.2	44.3	44.5	42.7	42.5	40.0
West	18.8	19.5	19.9	20.7	19.1	21.7	22.5	28.0
Midwest	19.6	18.6	19.0	17.6	17.9	19.1	18.8	15.3
Dual-eligible for Medicaid	37.8	37.3	38.6	40.4	42.1	43.6	44.3	39.8
hdIIV								

**Characteristic**	**2014/15**	**2015/16**	**2016/17**	**2017/18**	**2018/19**	**2019/20**	**2020/21**	**2021/22**
No. of individuals	3347	4212	25,152	61,340	68,248	71,528	74,459	54,293
Age, mean (SD)	75.8 (7.0)	75.7 (7.0)	74.7 (7.0)	74.5 (7.0)	74.5 (7.0)	74.4 (7.0)	74.3 (7.0)	74.7 (7.0)
Male	53.3	54.3	51.8	51.7	51.6	51.9	52.1	53.1
Race								
White	73.3	72.2	64.7	62.2	62.7	62.5	62.4	64.2
Black	20.6	21.4	29.0	30.7	29.8	30.1	29.9	28.0
Other	6.1	6.4	6.4	7.0	7.5	7.5	7.7	7.8
Residence in metropolitan statistical area	86.7	87.3	86.4	88.0	87.9	87.5	88.1	88.0
Region								
Northeast	19.1	18.7	18.0	16.6	16.6	15.9	15.9	17.1
South	36.1	33.6	44.5	44.3	43.9	45.3	45.0	42.8
West	14.4	14.9	17.2	19.1	20.2	19.5	19.9	19.9
Midwest	30.4	32.8	20.3	20.0	19.3	19.2	19.2	20.2
Dual-eligible for Medicaid	26.1	24.3	32.0	35.9	36.4	36.7	36.3	32.5
ccIIV								
No. of individuals	564	367	306	1924	4007	4235	4138	2662
Age, mean (SD)	75.5 (7.3)	75.3 (7.2)	74.9 (7.5)	74.3 (7.4)	74.3 (7.2)	74.5 (7.3)	74.4 (7.2)	74.9 (7.2)
Male	52.3	46.6	51.0	50.4	51.3	51.4	52.7	51.8

**Characteristic**	**2014/15**	**2015/16**	**2016/17**	**2017/18**	**2018/19**	**2019/20**	**2020/21**	**2021/22**
Race								
White	59.2	65.9	60.5	58.0	59.6	56.8	57.9	58.8
Black	22.3	20.4	32.0	31.1	32.2	32.4	31.3	30.5
Other	18.4	13.6	7.5	11.0	8.2	10.7	10.8	10.8
Residence in metropolitan statistical area	94.9	94.8	90.2	93.1	93.4	93.3	90.8	91.2
Region								
Northeast	21.4	38.4	38.2	23.9	31.0	28.4	29.5	36.9
South	14.4	28.3	25.8	38.4	39.8	38.5	39.3	36.4
West	52.3	14.7	23.5	25.0	15.8	17.8	16.7	15.0
Midwest	11.9	18.5	12.4	12.7	13.4	15.3	14.5	11.8
Dual-eligible for Medicaid	36.0	42.0	38.9	38.9	39.2	41.1	41.5	41.0
aIIV								
No. of individuals	<11*	<11*	178	793	1128	2128	3439	5087
Age, mean (SD)	<11*	<11*	75.8 (7.6)	75.1 (7.2)	75.3 (7.1)	75.3 (7.1)	75.4 (7.0)	75.5 (7.2)
Male	<11*	<11*	52.8	56.5	55.6	55.2	56.9	53.5
Race								
White	<11*	<11*	66.8	67.0	64.0	66.4	67.1	67.5
Black	<11*	<11*	28.6	27.9	26.1	24.5	24.3	19.3

**Characteristic**	**2014/15**	**2015/16**	**2016/17**	**2017/18**	**2018/19**	**2019/20**	**2020/21**	**2021/22**
Other	<11*	<11*	<11*	5.2	9.9	9.1	8.6	13.2
Residence in metropolitan statistical area	<11*	<11*	87.1	94.0	93.3	92.6	93.7	91.4
Region								
Northeast	<11*	<11*	15.2	20.0	21.5	32.6	28.4	18.6
South	<11*	<11*	51.7	49.6	45.6	38.2	38.7	32.9
West	<11*	<11*	11.8	14.4	19.1	14.7	18.1	31.7
Midwest	<11*	<11*	21.4	16.0	13.8	14.4	14.8	16.9
Dual-eligible for Medicaid	<11*	<11*	41.6	30.4	30.0	30.4	28.9	32.3
RIV								
No. of individuals	18	18	18	183	381	793	531	397
Age, mean (SD)	76.7 (6.7)	71.3 (7.2)	72.2 (5.8)	76.4 (7.4)	75.2 (7.1)	74.9 (7.0)	74.8 (7.3)	74.5 (6.9)
Male	<11*	<11*	<11*	51.4	53.8	54.7	57.1	56.2
Race								
White	66.7	<11*	72.2	72.1	69.0	64.1	67.8	71.5
Black	<11*	<11*	<11*	<11*	24.9	30.4	26.6	22.4
Other	<11*	<11*	<11*	<11*	6.0	5.6	5.7	6.0
Residence in metropolitan statistical area	<11*	<11*	<11*	73.8	76.6	93.2	88.7	92.2

Characteristic	2014/15	2015/16	2016/17	2017/18	2018/19	2019/20	2020/21	2021/22
Region								
Northeast	<11*	<11*	<11*	44.3	24.4	27.6	38.4	31.0
South	<11*	66.7	<11*	<11*	45.4	50.4	30.7	44.6
West	<11*	<11*	<11*	<11*	4.2	12.2	19.4	11.3
Midwest	<11*	<11*	<11*	37.7	26.0	9.7	11.5	13.1
Dual-eligible for Medicaid	<11*	<11*	<11*	22.4	32.3	30.0	34.5	27.7
Unvaccinated								
No. of individuals	18,176	18,099	22,160	17,915	16,969	17,319	14,812	15,374
Age, mean (SD)	74.5 (7.1)	74.4 (7.1)	74.3 (7.1)	74.4 (7.1)	74.1 (7.0)	74.2 (7.0)	74.2 (7.0)	74.6 (7.0)
Male	47.9	47.8	49.1	49.2	49.7	50.1	50.0	50.5
Race								
White	54.5	54.9	55.2	55.9	55.6	56.1	56.8	59.8
Black	39.8	39.4	38.9	38.3	38.3	37.2	36.4	33.1
Other	5.7	5.7	5.9	5.8	6.1	6.7	6.7	7.1
Residence in metropolitan statistical area	89.0	89.2	89.6	89.0	89.6	90.0	88.8	88.8
Region								
Northeast	20.0	20.7	20.2	20.6	19.6	20.5	18.6	18.3
South	42.4	41.9	42.5	42.7	43.8	42.7	43.9	42.2

**Characteristic**	**2014/15**	**2015/16**	**2016/17**	**2017/18**	**2018/19**	**2019/20**	**2020/21**	**2021/22**
West	17.1	17.3	17.7	17.2	17.9	19.5	20.1	21.1
Midwest	20.5	20.1	19.6	19.4	18.8	17.3	17.3	18.4
Dual-eligible for Medicaid	41.4	40.7	40.3	40.6	41.7	41.8	41.9	39.6

Abbreviations: aIIV, adjuvanted inactivated influenza vaccine; ccIIV cell culture-based inactivated influenza vaccine; hdIIV, high dose inactivated influenza vaccine; RIV, recombinant influenza vaccine; SD, standard deviation; sdeIIV, standard dose, egg-based inactivated influenza vaccine.

* Percentages based on frequencies <11 suppressed following USRDS publication guidelines.
